# Comment on: The Vitamin D–Folate Hypothesis as an Evolutionary Model for Skin Pigmentation: An Update and Integration of Current Ideas, *Nutrients* 2018, *10*, 554

**DOI:** 10.3390/nu10111753

**Published:** 2018-11-14

**Authors:** Peter M. Elias, Mary L. Williams

**Affiliations:** Department of Dermatology, University of California San Francisco and Veterans Affairs Medical Center, 4150 Clement Street, San Francisco, CA 94121, USA; elias.williams1@gmail.com

**Keywords:** Evolution, folic acid, pigmentation, skin, ultraviolet irradiation, vitamin D

Dear Editor,

In response to a recent article by Jones et al. (Nutrients 10: 554–568, 2018) [1], we agree that three distinctive features evolved in *Homo erectus* prior to the emergence of modern humans. These include the widespread dispersal of eccrine glands; a dramatic reduction in furry pelage, followed by dense pigmentation of recently exposed skin. It is now widely accepted that the first two events evolved either in close succession or concurrently in order to facilitate heat dispersion as hominins ventured onto torrid, dry open savannahs in response to sustained megadroughts that fragmented and shrank tropical forests [2].

Yet, the basis for the subsequent development of deep cutaneous pigmentation continues to provoke intense debate. Though no one doubts that cutaneous pigmentation evolved in response to exposure to potentially toxic levels of ultraviolet B irradiation (UVB) in equatorial Africa [3], the contentious issue is “why.” Jones et al. take issue with our proposition that pigmentation evolved to optimize permeability and antimicrobial barrier function in the hostile, infectious soup of equatorial Africa [4,5,6]. This hypothesis is based upon our prior descriptions of multiple functional advantages endowed upon darkly vs. lightly pigmented humans and mice [7,8]. However, Jones et al. dispute our conclusion, based upon earlier studies that showed a less-efficient barrier in African-Americans. But our data were based upon differences in degrees of pigmentation across several different ethnic groups—not upon racial categories—a distinction that should be abandoned, in any case, because of the large genetic variations among humans of different ethnicities, as well as the omnipresent risk of racial stereotyping.

Aside from the skin barrier concept, two other hypotheses continue to be advanced to explain the development of dense cutaneous pigmentation: first, to prevent the development of skin cancers—the so-called ‘genotoxic hypothesis’ [9]; and second, the possibility that skin darkened to prevent the photodegradation of folic acid and its biologically active metabolites [3,10]. We do not dispute the notion that folic acid deficiency occasionally results in severe congenital anomalies (e.g., neural tube defects, such as spina bifida) that could impair reproductive success. We also are fully aware that folic acid, its metabolites, and its regulatory genes can be readily degraded in vitro by UV irradiation [11]. Moreover, Jones’ group has identified latitude-dependent and UV-sensitive differences in the expression of several genes that regulate folic acid metabolism (cited in [12]), which they cite as further evidence in favor of the folic acid hypothesis. Jones et al. rely upon such results to defend the possibility that dense pigmentation evolved in the ultraviolet-drenched environs of Sub-Saharan Africa to prevent degradation of this critical nutrient. 

However, all of these data are correlative, and inconsistent with an expanding body of evidence against the folic acid hypothesis. Bolstered by the benefit of knowledge of skin structure and function, as well as the field of clinical dermatology, we summarize here the key arguments against the folic acid hypothesis: (1) The lack of direct evidence that incident UV irradiation reduces circulating folic acid levels. (2) In contrast to test tube studies, the fact that very little UV-B can reach the depths of the skin where folic acid and its metabolites circulate [13]. In an attempt to rescue this concept, it has been noted that UV-A sensitizers in the blood, such as riboflavin, could amplify UV-induced photodegradation [14], though the huge excess of circulating albumen and bilirubin would likely dampen any such risk of photosensitization [15]. (3) The very low incidence of congenital anomalies serious enough to interfere with reproductive fitness, even in populations that exhibit subnormal folic acid levels [16,17]. (4) The observation that neural tube defects are much more common in some northern countries (i.e., the United Kingdom and Ireland—1 in 100–400 births) in comparison to more southern latitudes (continental Europe and Latin America—0.75 in 100,000 births). Thus, neural tube defects can result from causes other than folic acid deficiency. (5) The ready bioavailability of folic acid from multiple dietary sources that were widely available in Sub-Saharan Africa when pigmentation evolved. (6) Most importantly, there is *direct* (as opposed to correlative) evidence that UV-B and UV-A, even when repeatedly administered and at supraphysiologic doses for the therapy of inflammatory dermatoses, such as psoriasis, do not reduce circulatory folic acid levels [18,19,20]. In light of the above, as well as further evidence against the folic acid hypothesis presented in [21], we respectfully submit that the folic acid hypothesis is simply untenable.

We would also note that the vitamin D corollary of the “yin-yang” hypothesis, as presented by Jones et al. (i.e., that pigment dilution occurred in response to a need to bolster cutaneous vitamin D production as modern humans emigrated from Africa into Europe), is equally flawed. A re-examination of the famed “Loomis diagram” [22] shows that, with the exception of humans who migrated behind retreating glaciers to the far north of Europe, only moderate pigment dilution occurred everywhere else in Eurasia. For example, Chinese and Japanese family members, residing at widely separated latitudes, exhibit comparable degrees of pigmentation (cited in [6]). Moreover, circulating vitamin D levels are higher in northern Europeans than in central and southern Europeans [23]. Finally, how can Jones et al. reconcile several recent population genetic studies which have demonstrated the persistence of dark pigmentation in Mesolithic populations of southern, central, and northern Europeans as recently as 7 thousand years ago (e.g., [24])? Indeed, even in the far North, light pigmentation evolved only in the last 5000 years—and not during 30,000+ years that humans had occupied Eurasia [25]. Note also that rickets appeared only after the rise of the Industrial Age, when pollution darkened European skies, and that osteoporosis is less common in African-American people then in people of northern European extraction (see [4,5,6,21]). These observations support recent studies showing that, rather than changes in pigmentation genes, nonpigment-related mutations have evolved recently to enhance vitamin D production in northern Europeans. These include: loss-of-function mutations in the epidermal structural protein; filaggrin [23], which is hydrolyzed into the most potent UV-B absorber in the skin, trans-urocanic acid; and the gene encoding 7-dehydrocholesterol reductase, which would inevitably result in elevated levels of 7-dehydrocholesterol, the immediate sterol precursor of previtamin D3 [26]. 

Yet, a moderate degree of skin lightening did occur across Eurasia and southcentral Europe. We proposed that, rather than to enhance vitamin D3 production, evolution favored mutations, such as polymorphisms in the melanocortin receptor type 1 (MC1R), because dense pigmentation was no longer necessary in light of less stringent barrier requirements. This well-known concept in clinical medicine is called ‘metabolic conservation,’ allowing the diversion of precious calories towards more urgent priorities [6].

Finally, one would anticipate that a molecule as important as vitamin D would be subject to well-designed biological checks and balances, and melanin would be a relatively crude way to control such a critical process. Changes in melanin production by melanocytes and delivery to its destination in epidermal keratinocytes occur slowly—not in a timely enough fashion to titrate UV absorption on a minute-by-minute basis. We therefore conclude that there is little or no credible evidence that pigment dilution evolved in order to bolster cutaneous vitamin D production.
